# *Tyro3/Axl/Mertk*-deficient mice develop bone marrow edema which is an early pathological marker in rheumatoid arthritis

**DOI:** 10.1371/journal.pone.0205902

**Published:** 2018-10-18

**Authors:** Claire E. J. Waterborg, Marije I. Koenders, Peter L. E. M. van Lent, Peter M. van der Kraan, Fons A. J. van de Loo

**Affiliations:** Experimental Rheumatology, Department of Rheumatology, Radboud Institute for Molecular Life Sciences, Radboud University Medical Center, Nijmegen, the Netherlands; Institut d'Investigacions Biomediques de Barcelona, SPAIN

## Abstract

Rheumatoid arthritis is an auto-immune disease of the synovial joints, hallmarked by chronic inflammation and subsequent progressive tissue destruction. TYRO3, AXL and MER (gene name *Mertk*) (TAM) receptors are part of a negative feedback signaling system in the immune reaction and mediate efferocytosis thereby tempering the inflammatory process. We have shown that *Axl*^*-/-*^ and *Mertk*^*-/-*^ mice develop more severe arthritis whereas activating these receptors by overexpressing their ligands *Pros1* and *Gas6* ameliorates arthritis. Mice genetically ablated for the three genes of the TAM receptor family *Tyro3*/*Axl*/*Mertk* (TAM triple knock-out or TKO) have been described to spontaneously develop macroscopic signs of arthritis. In this study we aimed to analyze arthritis development in TAM TKO mice histologically to determine the extent and sequence of pathological changes in the joint. Ankle joints of three different age groups, adolescence (14 weeks), mature adult (34 weeks) and middle-age (52 weeks), of TAM TKO or wild-type mice were examined macroscopically, histologically and immunohistochemically. Surprisingly, until the age of 52 weeks, none of the mice examined developed spontaneous macroscopic signs of arthritis. There was no synovial inflammation nor any signs of damage to the cartilage or bone. However, bone marrow edema was observed in TAM TKO mice in the two latter age groups. The infiltrate in the bone marrow was characterized by both myeloid cells and lymphocytes. This study showed that TAM TKO mice developed a pre-stage (pre-clinical phase) of arthritis marked by bone marrow edema.

## Introduction

Rheumatoid arthritis (RA) is an auto-immune disease marked by chronic and unrestrained inflammation, often in multiple synovial joints in the same patient. In the arthritic joint, the synovium is infiltrated by both innate and adaptive immune cells which, together with the proliferation of the tissue-resident fibroblasts, leads to pannus tissue formation at the articular cartilage and bone interface. Overall, this eventually leads to damage and loss of the articular cartilage matrix and bone tissue [1, 2].

One family of receptors that has been linked to RA and experimental models of RA are the TAM receptors [3–10]. The TAM receptors–TYRO3, AXL and MER (gene name *Mertk*)–are a family of tyrosine kinases essential for immune homeostasis by negatively regulating the innate immune response. Together with their ligands Growth Arrest Specific 6 (GAS6) and Protein S (PROS1), they also mediate the resolution of inflammation by facilitating efferocytosis [11]. We have found that both AXL and MER play a suppressive role by alleviating joint inflammation and others have found that TYRO3 plays an enhancing role in bone degradation and synovial hyperplasia in experimental arthritis [5, 7, 10] (manuscript under review).

More than a decade ago, it was reported that mice lacking all three TAM receptors (triple knock-out or TKO) develop a lymphoproliferative disorder accompanied by broad-spectrum autoimmunity including splenomegaly [12]. In this paper, they showed TAM TKO mice spontaneously developed clinical characteristics of RA including swollen footpads in a 6-month-old mouse. Furthermore, deficiency of all three receptors led to enhanced proliferation of both B cells and T cells. Moreover, auto-antibodies were detectable in serum of these mice.

In this study, we aimed to analyze arthritis development in TAM TKO mice histologically to determine the extent and sequence of pathological changes in the disease-prone ankle joints. Surprisingly, until the age of 52 weeks, none of the TAM TKO mice examined developed any macroscopic signs of arthritis. Histologically, neither tissue destruction nor synovial inflammation was observed. However, bone marrow edema (BME), which was characterized by an infiltrate of myeloid cells and lymphocytes, was present at the age of 34 weeks which had persisted to the age of 52 weeks.

## Materials and methods

### Mice

*Tyro3*/*Axl*/*Mertk*-deficient mice were generated as described previously [13]. Mice were bred and housed in the Salk Institute Animal Facility under a 12 hour light/dark cycle in individually-ventilated cages, with freely available food and water. Mice were on a mixed C57BL/6 × 129/Sv background. Both male and female mice were used, and wild-type (WT) and *Tyro3*/*Axl*/*Mertk*-deficient mice were sex-matched. At the ages of 14 weeks (n = 6), 34 weeks (n = 6) and 52 weeks (n = 12) (adolescent, mature adult and middle-age, respectively), mice were sacrificed by an overdose of isoflurane. As the mice were not visibly affected and suffering by the genotype, no analgesics were used to alleviate suffering. All procedures were approved by and conducted according to guidelines established by the Salk Institutional Animal Care and Use Committee (IACUC).

### Histology

Whole ankle joints were dissected, fixed in 4% phosphate-buffered formalin, followed by decalcification with 5% formic acid for 7 days. Tissues were dehydrated with an automated tissue processing apparatus (Leica ASP 300, Leica Biosystems) and embedded in paraffin. Semi-serial tissue sections (7 μm) were stained with hematoxylin and eosin (H&E) (both Merck), safranin O and fast green (both Brunschwig Chemie) or were used for immunohistochemical staining.

### Quantification of synovial cellularity and BME

**Fig 1:**
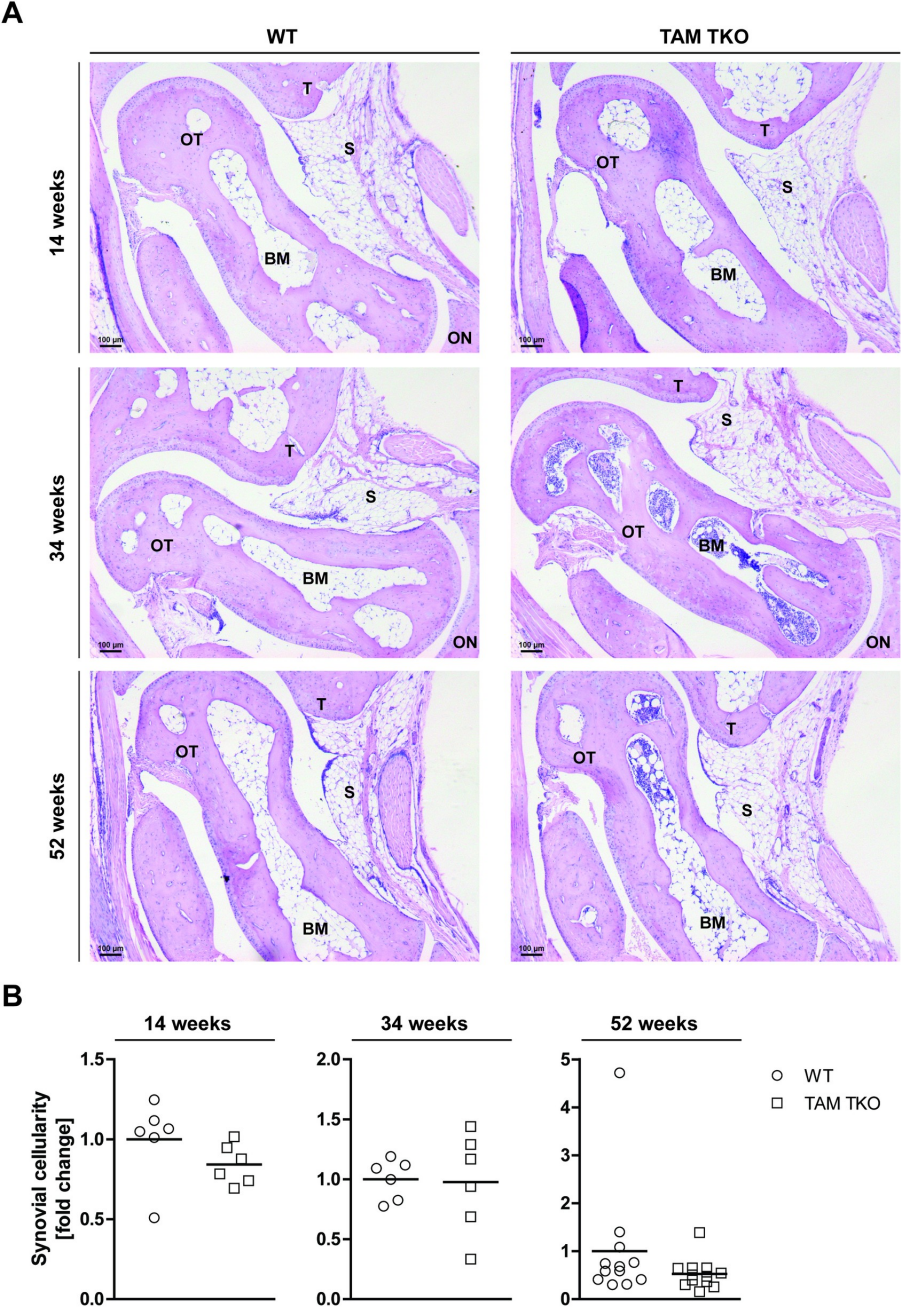
Effect of genetic deletion of *Tyro3*/*Axl/Mertk* on synovial cellularity in ankle joints. (A) Ankle joints of naive wild-type (WT) and *Tyro3*/*Axl/Mertk* triple knock-out (TAM TKO) mice of 14, 34 and 52 weeks old were processed for histology. Sections were stained with hematoxylin and eosin. Shown are representative pictures in 50x magnification. Staining is representative for 6 mice (14 and 34 weeks old mice) or 12 mice (52 weeks old mice). (B) Synovial cellularity was quantified in all joints in a random and blinded manner using Leica Application Suite software. Data are presented as dot-plots with mean. Data were statistically analyzed with a Mann Whitney test. n = 6 at 14 and 34 weeks old, n = 12 at 52 weeks old. BM = bone marrow, ON = os naviculare, OT = os talus, S = synovium, T = tibia. Black scale bars represent 100 μm.

Histological slides of ankle joints stained with H&E were blinded and randomized. For randomization, each joint was given a random number. Pictures of the indicated region of the ankle joint were taken with the Leica DMR microscope (Leica Microsystems) at 50x magnification. To measure the synovial cellularity, the surface area of the synovium was selected as the region of interest. The surface area staining positive for H&E was measured and corrected for the total area that was measured. To measure BME, the surface area of the bone marrow cavity was selected as the region of interest. The surface area staining positive for H&E was measured and corrected for the total area (bone marrow cavity) that was measured. Two to three tissue sections were examined per ankle joint per region (tibia, os talus or os naviculare), the average of two to three sections per region was calculated and is depicted. For all measurements, Leica Application Suite software (Leica Microsystems) was used. WT controls were set at 1 in Figs 1 and 3. WT controls of different ages were compared in S1 Fig and area over total area, calculated as percentage, is depicted

### Quantification of articular cartilage depletion

Histological slides of ankle joints stained with safranin O and fast green were blinded and randomized. For randomization, each joint was given a random number. Pictures of the indicated region of the ankle joint were taken with the Leica DMR microscope (Leica Microsystems) at 100x magnification. Two to three tissue sections were examined per ankle joint per region, the average per joint was calculated and is depicted. To determine cartilage depletion, mean blue was measured using Leica Application Suite software (Leica Microsystems). The surface area of the cartilage was selected and the amount of blue light passing through the region of interest was measured by the software. WT controls were set at 1.

### Immunohistochemistry

Protein expression was evaluated on 7 μm tissue sections of ankle joints. Sections were deparaffinized and rehydrated. Endogenous peroxidase was blocked by 3% (v/v) hydrogen peroxide in methanol for 20 minutes at room temperature (RT). Sections were washed in between incubation steps with phosphate-buffered saline (PBS) 3 times for 5 minutes. For all sections, a biotin-streptavidin horseradish peroxidase detection system was used according to manufacturer’s protocol (Vector Laboratories; PK6101). Bound complexes were visualized with diaminobenzidine (DAB) (Merck) for 10 minutes at RT. Sections were counterstained with hematoxylin for 30 seconds. Sections were dehydrated and enclosed with Permount.

#### F4/80

For F4/80 detection, antigen-retrieval was performed in citrate buffer (pH 6.0) heated to 37ºC for 2 hours and subsequent trypsin treatment (Sigma; T-9201; 0.075% in PBS) at 37°C for 7 minutes. Sections were blocked by 10% (v/v) rabbit serum in PBS for 20 minutes at RT. Tissue sections were incubated with rat anti-mouse F4/80 (eBioscience; 14-4801-81; monoclonal (BM8); 1 μg/mL) or rat IgG2a (BD Pharmingen; 553927; 1 μg/mL) overnight at 4°C. Subsequently, sections were incubated with biotinylated rabbit anti-rat IgG (Vector Laboratories; BA-4001; 1:200) for 30 minutes at RT. Antibodies were diluted in 2% (v/v) rabbit serum in PBS.

#### CD3

For CD3 detection, antigen retrieval was performed in citrate buffer (pH 6.0) heated to 60ºC for 10 minutes. Sections were blocked by 5% (w/v) skimmed milk (ELK) 3% (v/v) fetal calf serum (FCS) 2% (w/v) bovine serum albumin (BSA) in PBS for 1 hour at RT. Tissue sections were incubated with rabbit anti-mouse CD3 (DAKO; A0452; polyclonal; 3 μg/mL) or rabbit IgG control (DAKO; X0936; 3 μg/mL) overnight at 4°C. Subsequently, sections were incubated with biotinylated goat anti-rabbit IgG (PK6101; 1:400) for 30 minutes at RT. Antibodies were diluted in 5% ELK 3% FCS 2% BSA in PBS.

#### CD19

For CD19 detection, antigen retrieval was performed in citrate buffer heated (pH 6.0) to 60ºC for 10 minutes. Sections were blocked by 5% ELK 3% FCS 2% BSA in PBS for 1 hour at RT. Tissue sections were incubated with rabbit anti-mouse CD19 (Abcam; ab203615; polyclonal; 0.4 μg/mL) or rabbit IgG control (DAKO; X0936; 0.4 μg/mL) overnight at 4°C. Subsequently, sections were incubated with biotinylated goat anti-rabbit IgG (PK6101; 1:400) for 30 minutes at RT. Antibodies were diluted in 5% ELK 3% FCS 2% BSA in PBS.

### Statistical analysis

Data were analyzed with GraphPad Prism software (version 5.03). Data are shown as dot-plots with mean. Analysis of significance was performed using a two-tailed Mann Whitney test. p-Values lower than 0.05 were considered to be significantly different.

## Results

### Synovial cellularity and connective tissue integrity is unaltered in the ankle joints of mice genetically ablated for *Tyro3*/*Axl/Mertk*

**Fig 2:**
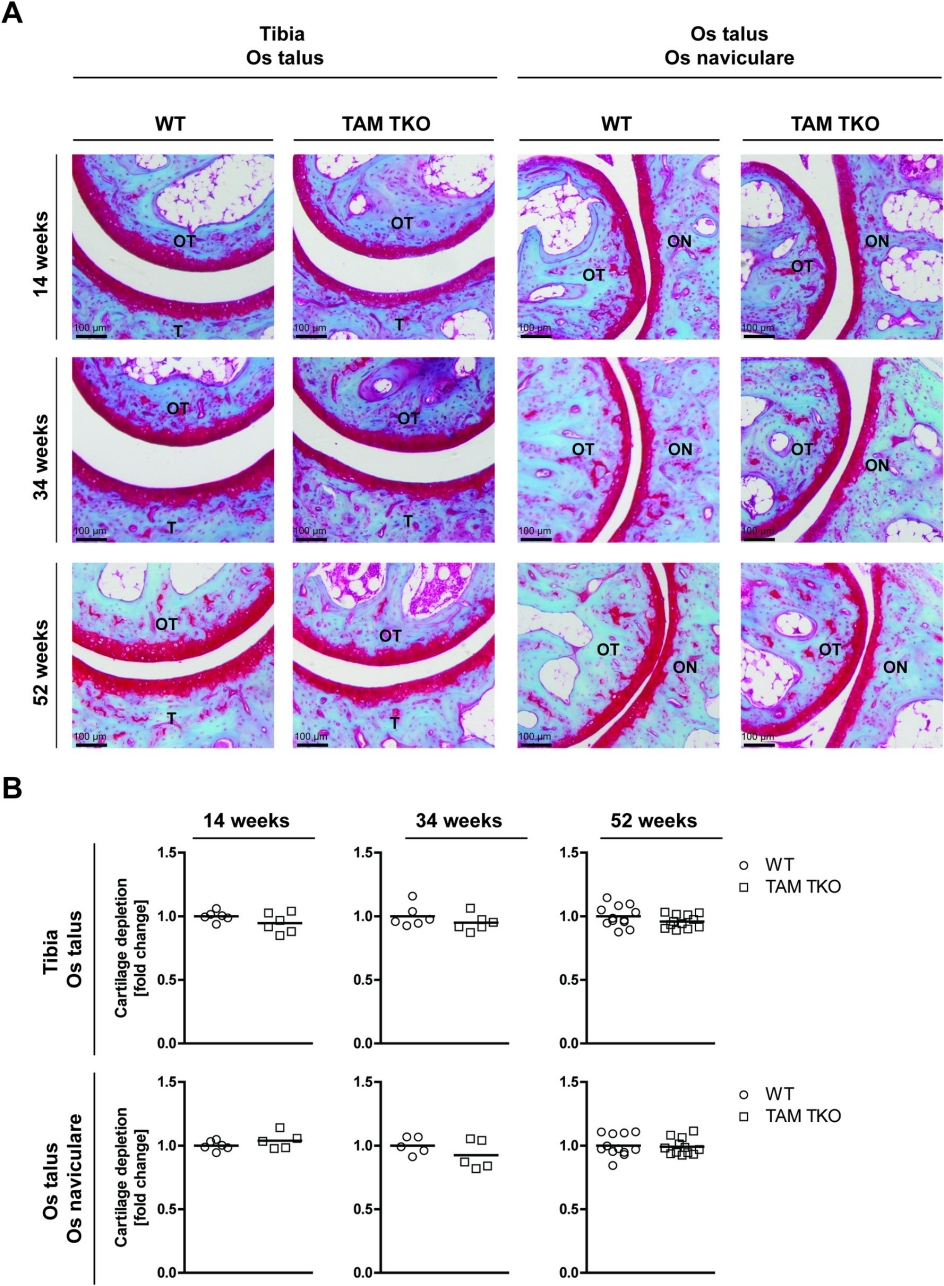
Effect of genetic deletion of *Tyro3*/*Axl/Mertk* on cartilage destruction in ankle joints. (A) Ankle joints of naive wild-type (WT) and *Tyro3*/*Axl/Mertk* triple knock-out (TAM TKO) mice of 14, 34 and 52 weeks old were processed for histology. Sections were stained with safranin O and fast green. Shown are representative pictures of the tibia–os talus region and os talus–os naviculare region in 50x magnification. Staining is representative for 6 mice (14 and 34 weeks old mice) or 12 mice (52 weeks old mice). (B) Cartilage proteoglycan depletion was quantified in both regions in all joints in a random and blinded manner using Leica Application Suite software. Data are presented as dot-plots with mean. Data were statistically analyzed with a Mann Whitney test. n = 6 at 14 and 34 weeks old, n = 12 at 52 weeks old. ON = os naviculare, OT = os talus, T = tibia. Black scale bars represent 100 μm.

We examined the ankle joints of mice deficient for *Tyro3*/*Axl/Mertk* at three different ages, namely adolescence (14 weeks), mature adult (34 weeks) and middle-age (52 weeks). Surprisingly, we found that TAM TKO mice up to 1 year of age did not develop any macroscopic arthritis-like symptoms, such as swollen toes or footpads, spontaneously. The ankle joint is the privileged site of disease in spontaneous arthritis models [14], but histological examination showed that the ankle joints in the TAM TKO mice appeared to be indistinguishable from their WT controls. The synovial cellularity was comparable between TAM TKO and WT mice at all three ages (Fig 1). Next, we examined the tissue destruction. No bone erosion was observed in any of the mice. In addition, no cartilage erosion was observed nor were there any prominent and significant differences on the upper-layer depletion of cartilage proteoglycan (loss of safranin O staining of the non-calcified upper cartilage layer) (Fig 2).

### TAM TKO mice develop BME in the ankle joint region

**Fig 3:**
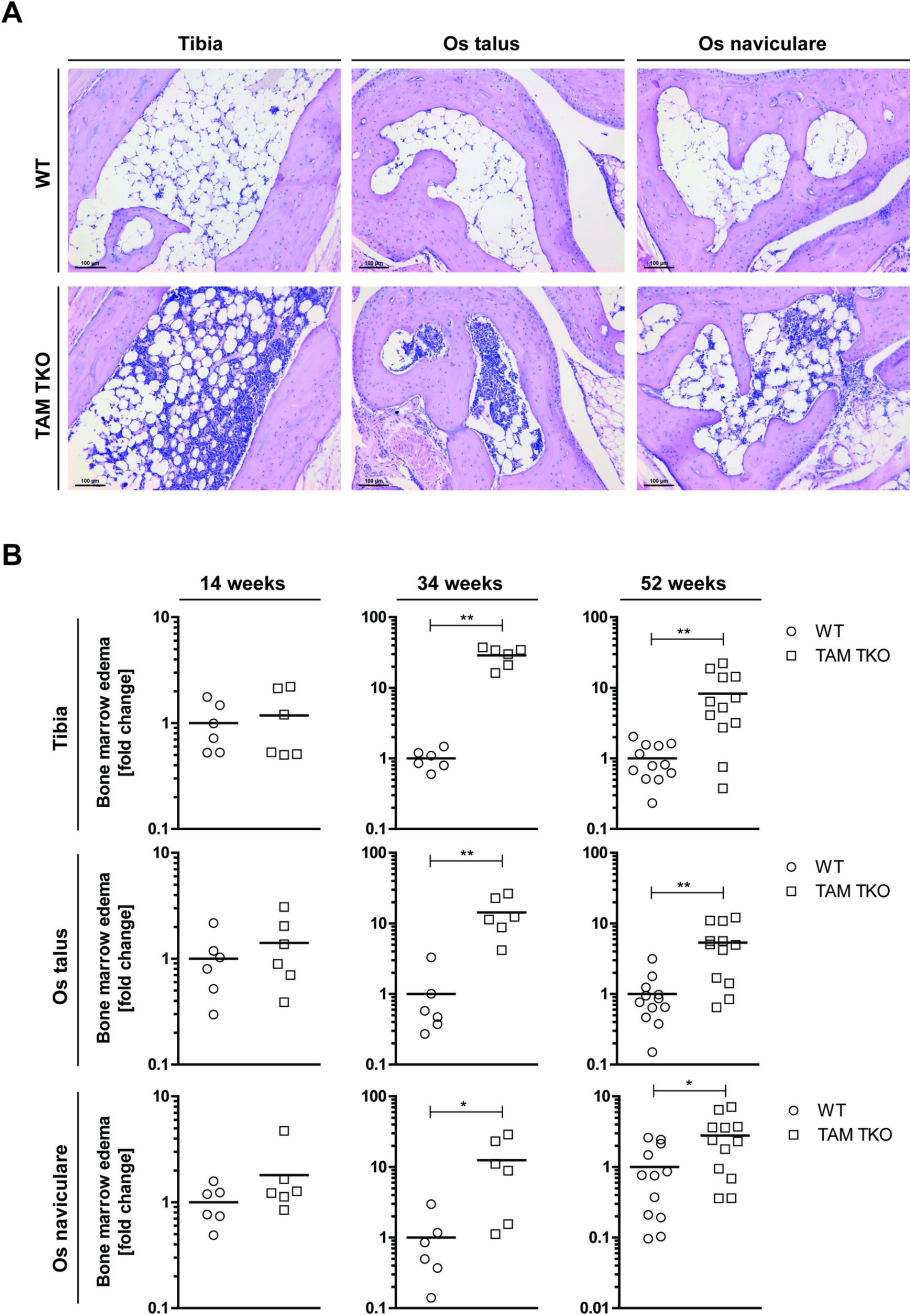
Effect of genetic deletion of *Tyro3*/*Axl/Mertk* on bone marrow edema. Ankle joints of naive wild-type (WT) and *Tyro3*/*Axl/Mertk* triple knock-out (TAM TKO) mice of 14, 34 and 52 weeks old were processed for histology. (A) Sections were stained with hematoxylin and eosin. Shown are representative pictures in 100x magnification of the os naviculare, os talus and tibia of 52 weeks old mice. Staining is representative for 12 mice. (B) Bone marrow edema was quantified in all joints in a random and blinded manner using Leica Application Suite software. Data are presented as dot-plots with mean. Data were statistically analyzed with a Mann Whitney test. * = p<0.05, ** = p<0.01. n = 6 at 14 and 34 weeks old, n = 12 at 52 weeks old. Black scale bars represent 100 μm. See S1 Fig for comparison of bone marrow edema quantification in WT mice among different ages.

No tissue destruction nor synovial inflammation was present in any of the joints examined in TAM TKO mice (Figs 1 and 2). Therefore, we focused on BME, a phenomenon which has been described to be present in the preclinical phase of RA [15, 16]. It appeared that TAM TKO mice developed an inflammatory infiltrate in the bone marrow replacing the fat-rich marrow, termed BME. Indeed, BME was enhanced in TAM TKO mice compared to WT in all three regions investigated in mature and middle-age mice (Fig 3). During aging, the fat-rich bone marrow composition in the WT mice was not changed (see S1 Fig). These data show that the TAM TKO mice examined developed early signs of arthritis without signs of joint pathology characteristic for full-blown arthritis.

### BME in TAM TKO mice is characterized by cells of myeloid and lymphocyte lineage

**Fig 4:**
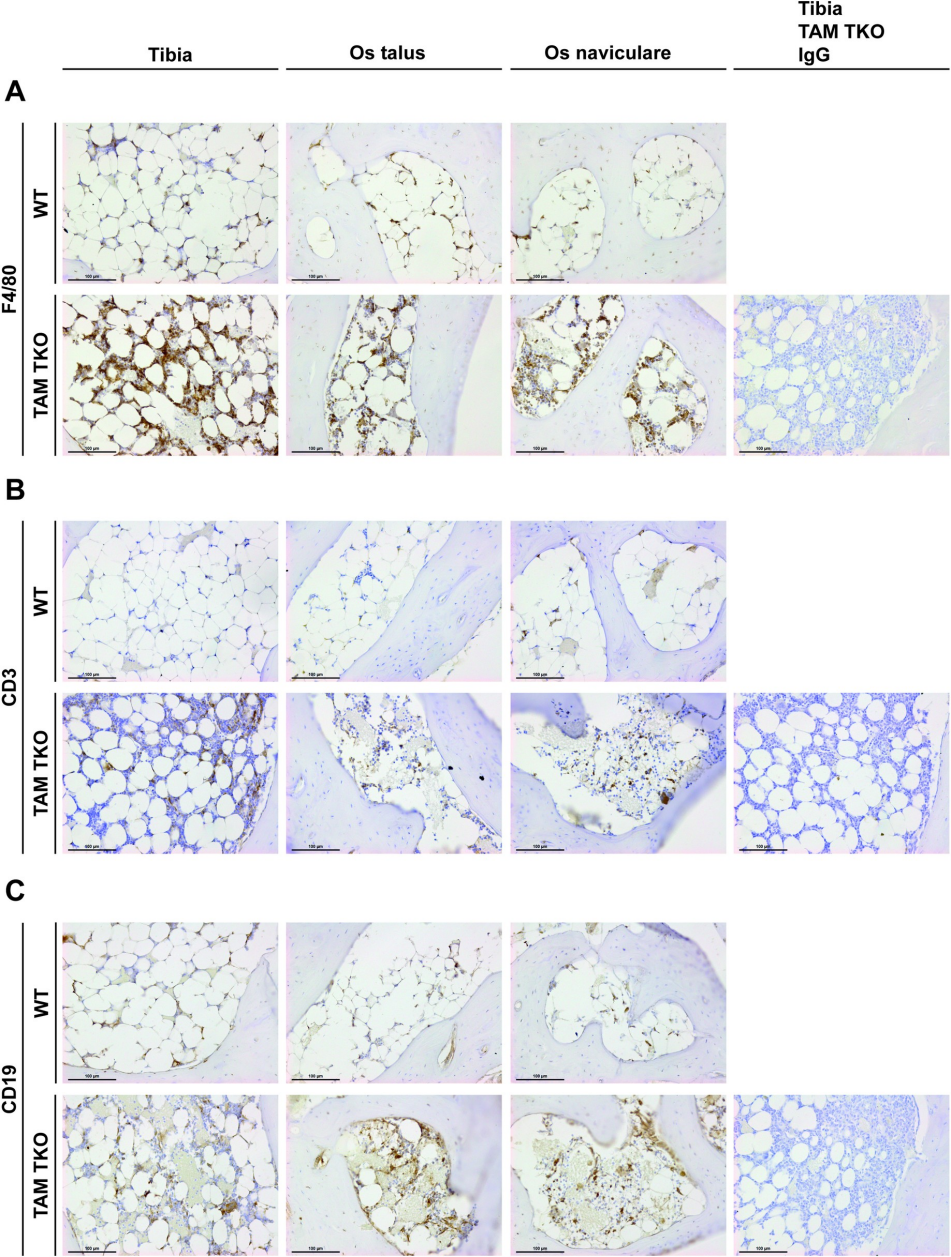
Characterization of bone marrow cells in mice lacking *Tyro3*/*Axl/Mertk*. Ankle joints of naive wild-type (WT) and *Tyro3*/*Axl/Mertk* triple knock-out (TAM TKO) mice of 52 weeks old were processed for histology. Sections were immunohistochemically stained for the cell surface marker of interest, or IgG negative control, and counterstained with hematoxylin. Shown are representative pictures in 200x magnification of the os naviculare, os talus and tibia. Staining is representative for 12 mice. (A) F4/80, (B) CD3, (C) CD19. Black scale bars represent 100 μm.

To further characterize the type of cells of the BME, cells were examined morphologically and cell surface markers were examined by immunohistochemistry. Although to a lesser extent than in WT mice, adipocytes were still present in the bone marrow of TAM TKO mice (Fig 3A, Fig 4). The infiltrate in the bone marrow of TAM TKO mice consisted of a significant amount of F4/80^+^ macrophages (Fig 4A). Moreover, cells of the lymphoid lineage were also abundantly present. CD20^+^ B cells, and to a lesser extent, CD3^+^ T cells were scattered between the F4/80^+^ cells (Fig 4B and 4C).

## Discussion

In this manuscript, we describe that mice deficient for *Tyro3*/*Axl*/*Mertk* do not develop clinical characteristics of arthritis until the age of 52 weeks. Nonetheless, they do develop an early phenomenon observed in RA, but which is not specific for RA, namely BME. However, in contrast to the publication in 2001 [12], they do not (any longer) develop any aberrant macroscopic signs of arthritis at mature adulthood. Moreover, histologically, joints are not characterized by synovial inflammation and lymphocyte invasion. This is remarkable as the source of the mice was from the same laboratory where they were initially described. Nevertheless, we cannot exclude that TAM TKO mice develop macroscopic signs of arthritis, synovial inflammation or connective tissue destruction at ages over 52 weeks.

In RA, the fat-rich tissue in the bone marrow, also called the yellow marrow, can be replaced by a cell-rich inflammatory environment. This phenomenon, called BME, strongly associates with development of bone erosions and poor functional outcome in patients with RA [17–21]. Very early in the disease process, BME is present and prognostic for the development from undifferentiated arthritis to RA [15, 16, 22]. Moreover, BME is the strongest predictor for bone erosions in RA patients using magnetic resonance imaging (MRI) [17–21]. Histological and immunohistochemical analysis showed that BME observed by MRI is characterized by an infiltrate of inflammatory cells into the bone marrow normally replaced by adipocytes upon ageing. The infiltrate mainly consists of lymphocytes, CD3^+^, CD4^+^, or CD8^+^ T cells and CD20^+^ B cells, but also CD68^+^ macrophages have been described [23–28]. One study in TNF-transgenic mice showed that the BME observed in these mice consisted mainly of myeloid cells [29]. We show that the BME observed in TAM TKO mice is also characterized by the presence of both lymphocytes and macrophages, the latter being abundantly present as quantified by the presence of the macrophage marker F4/80. This may confirm the lymphoproliferative disorder in the TAM TKO mice, caused by constitutively active cells of the myeloid lineage, as elegantly demonstrated by adoptive cell transfers [12]. It is unknown whether the lymphocytes in these mice were targeting any joint-specific antigens that could cause joint-specific disease.

It is tempting to speculate that the absence of spontaneous arthritis development we observed compared to the publication of Lu and Lemke [12], is a result of an interaction between genetic and environmental factors. Phenotypic differences in mice with similar genotypes, although from a different breeding generation, have been described before [30]. Moreover, long-term breeding of mouse lines results in marked changes in intestinal microbiota composition and microbiota can account for genotype-specific phenotypes [31]. This is in line with overall better hygiene and housing facilities of mice compared to a decade ago. Numerous studies implicate mucosal surfaces as sites of disease initiation in RA [32]. The simultaneous occurrence of periodontitis, a bacterial-induced inflammation of the gums, and RA suggests that oral pathogens may initiate the production of disease-specific antibodies against citrullinated proteins (ACPA) [33]. This coincides with the fact that periodontitis is characterized by the presence of similar citrullinated auto-antigens in neutrophils, caused by the bacteria *Aggregatibacter actinomycetemcomitans*, as found in the arthritic joint [34]. Interestingly, the TAM receptor ligand GAS6 has been shown to be an immunological regulator in oral mucosal homeostasis [35].

Currently, it remains to be elucidated what causes BME development in TAM TKO mice. We have shown that MER mediates efferocytosis in the arthritic joint [10]. Moreover, AXL plays a protective role in experimental arthritis, possibly by regulating the post-translational activation of IL-1β (manuscript accepted for publication). However, none of the joints examined in the single knock-out mice for *Axl* and *Mertk* showed any signs of BME, nor did the *Axl*/*Mertk* double knock-out (own observations). Therefore, it appears that all 3 receptors need to be knocked-out in order to develop this phenotype. As we found BME in mature TAM TKO mice, and this is a phenomenon present in preclinical RA, it is interesting to investigate whether it would be effective to treat these patients with GAS6 in a preclinical stage to stop the progression of BME and subsequent bone erosions. To conclude, TAM TKO mice develop and early stage of RA, namely BME, without spontaneously developing clinical and histopathological signs of arthritis.

## Supporting information

S1 Fig Effect of aging on bone marrow edema. Ankle joints of naive wild-type mice of 14, 34 and 52 weeks old were processed for histology. Sections were stained with hematoxylin and eosin. Bone marrow edema was quantified in the os naviculare, os talus and tibia in a random and blinded manner using Leica Application Suite software. Data are presented as dot-plots with mean. n = 6 at 14 and 34 weeks old, n = 12 at 52 weeks old. See Fig 3 for comparison of bone marrow edema quantification between wild-type and *Tyro3*/*Axl/Mertk* triple knock-out mice. These data are the same data as in Fig 3 for the wild-type mice, but presented in a different manner, to show the effect of aging on bone marrow edema.
(TIF)
