# Supplementary figures and images for: Tyro3/Axl/Mertk-deficient mice develop bone marrow edema which is an early pathological marker in rheumatoid arthritis
Source: PLoS One. 2018 Oct 18;13(10):e0205902. doi: 10.1371/journal.pone.0205902 (PMC6193696; doi:10.1371/journal.pone.0205902)

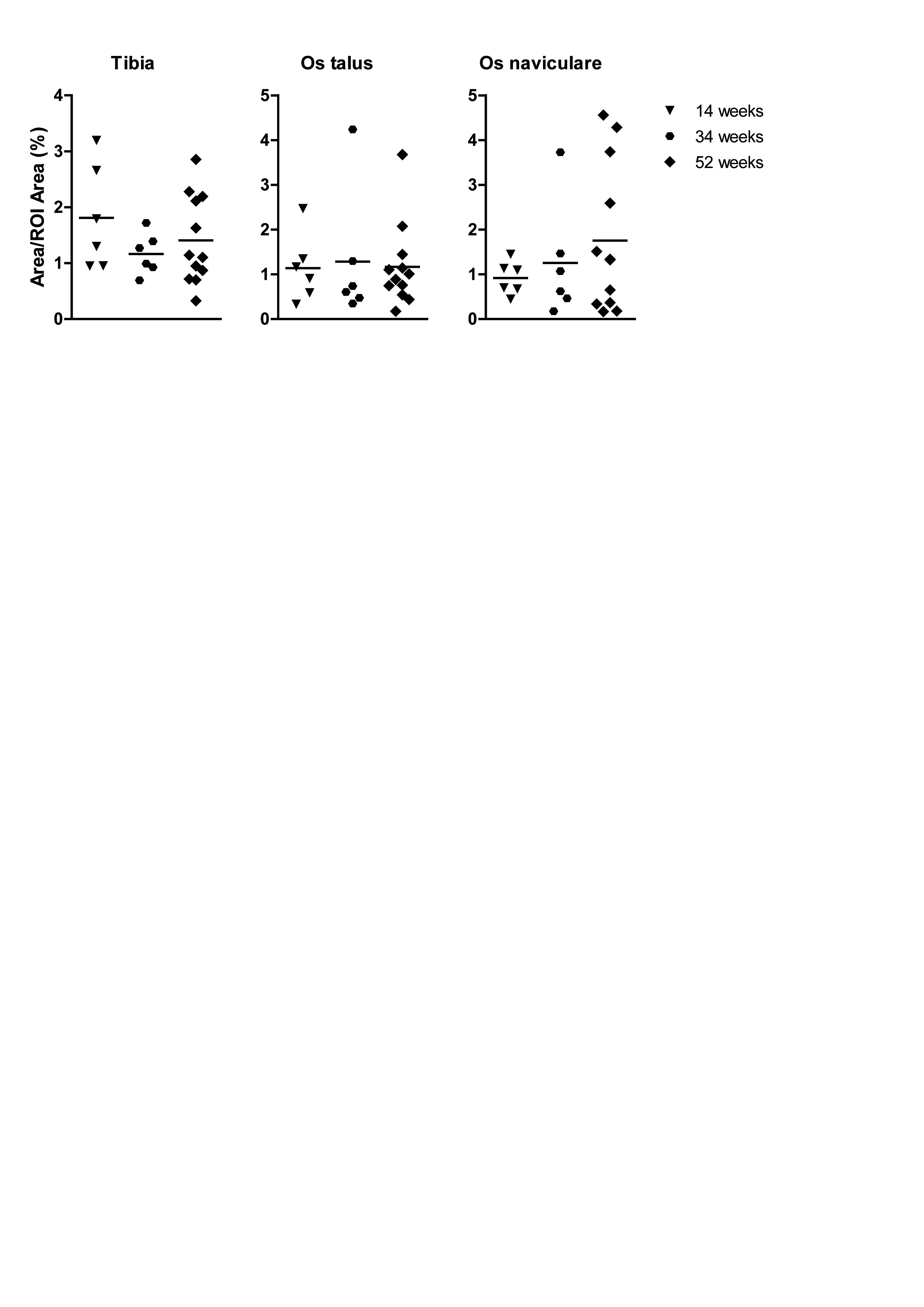

Supplement: S1 Fig — Ankle joints of naive wild-type mice of 14, 34 and 52 weeks old were processed for histology. Sections were stained with hematoxylin and eosin. Bone marrow edema was quantified in the os naviculare, os talus and tibia in a random and blinded manner using Leica Application Suite software. Data are presented as dot-plots with mean. n = 6 at 14 and 34 weeks old, n = 12 at 52 weeks old. See Fig 3 for comparison of bone marrow edema quantification between wild-type and Tyro3/Axl/Mertk triple knock-out mice. These data are the same data as in Fig 3 for the wild-type mice, but presented in a different manner, to show the effect of aging on bone marrow edema. (TIF) [file pone.0205902.s001.tif]
